# Editorial: Feeding difficulties in newborn infants and new approaches in practice

**DOI:** 10.3389/fped.2024.1462493

**Published:** 2024-09-05

**Authors:** Deniz Anuk Ince, Sahin Takci, Hasan Kilicdag, Ozden Turan

**Affiliations:** ^1^ Division of Neonatology, Department of Pediatrics, Baskent University Faculty of Medicine, Ankara, Türkiye; ^2^ Division of Neonatology, Department of Pediatrics, Ondokuz Mayıs University Faculty of Medicine, Samsun, Türkiye

**Keywords:** feeding, feeding difficulties, preterm, term infants, nutrition

**Editorial on the Research Topic**
Feeding difficulties in newborn infants and new approaches in practice

Feeding difficulties encompass a broad spectrum of disorders, including oral feeding problems, sucking-swallowing-respiration (SSR) incoordination, neurological and respiratory disorders, which are frequently observed in preterm infants due to immaturity. The prevalence of feeding difficulties is significantly higher in preterm infants, especially proportional to the degree of prematurity, compared to full-term infants (1). To provide targeted diagnostic and therapeutic support, it is essential to categorize feeding problems based on their underlying etiologies.

In preterm infants born before 32 weeks of gestation during their stay in the neonatal intensive care unit (NICU), several morbidities such as respiratory diseases (respiratory distress syndrome and bronchopulmonary dysplasia), brain injury (intraventricular hemorrhage, periventricular leukomalacia, or hypoxic injury), necrotizing enterocolitis, surgeries, and underlying craniofacial abnormalities may affect the normal development of sucking and swallowing (2). Additionally, the risk of oral feeding problems increases in infants with genetic syndromes, congenital deformities of the oropharyngeal structures, congenital heart diseases, surgical conditions and maternal metabolic conditions such as hypertension, diabetes, and drug use (2).

An estimated 80% of preterm infants experience difficulty with oral feeding during NICU hospitalization (3, 4). The introduction of oral feeding occurs when the infant demonstrates signs of oral feeding readiness in the NICU, typically occurs around 34 weeks postmenstrual age (5, 6). Pineda et al. (7) showed that preterm infants born ≤32 weeks gestation at term-equivalent age had lower Neonatal Eating Outcome Assessment scores and were more likely to exhibit tongue positioning, SSR incoordination, inadequate sucking bursts, the jaw and tongue discoordination during sucking, and difficulty completing the feeding compared with term infants. These immaturity-related conditions, especially in very preterm infants, often result in feeding intolerance or other feeding problems during the transition to oral feeding.

Feeding intolerance is a major cause of morbidity in preterm infants, leading to prolonged need for parenteral nutrition and associated complications such as sepsis, cholestasis, prolonged hospital stay, malnutrition, and poor neurodevelopmental outcomes (8). Ifran et al. developed a novel scoring system based on clinical symptoms and ultrasonographic findings to assess feeding intolerance in very preterm infants. The system includes criteria such as abdominal distention, hemorrhagic gastric fluid, intestinal peristaltic movement ≤18 per 2 min, gastric fluid residue >25%, and a resistive index >0.785. Color Doppler ultrasound of the superior mesenteric artery (SMA) was used to assess peak systolic velocity (PSV) and end-diastolic velocity (EDV). The resistive index (RI) was calculated using the formula (PSV—EDV)/PSV, which was automatically computed by the ultrasound machine. In evaluating SMA blood flow, both PSV and RI are commonly used ultrasound indicators. This study found that RI was an independent predictor of feeding intolerance. The scoring system has two advantages: ease of use and safety. Using this scoring system, clinicians can design feeding protocols at risk of feeding intolerance and may prevent long-term complications related to parenteral nutrition. A score of 5 or higher was identified as the cut-off level for discontinuing oral feeding (Ifran et al.).

Feeding difficulties often lead to prolonged hospitalization in NICUs. Immaturity of the gastrointestinal tract, reduced gastrointestinal motility, increased susceptibility to necrotizing enterocolitis, and other prematurity-related co-morbidities are the barriers to the transition to enteral feeding (9, 10). Early preterm infants may face delays in transition to full oral feeding due to insufficient sucking and SSR coordination and weak oropharyngeal musculature. Balcı et al. investigated the impact of oromotor exercise therapy on early preterm infants in the NICU, focusing on their transition to breastfeeding and improvement of feeding skills in the presence of feeding intolerance. Oromotor therapy was applied to early preterm infants for a month, three times a week, including non-nutritive sucking, massage of swallowing muscles and stimulation of sucking. The feeding skills were evaluated by Early Feeding Skills Assessment Tool and Preterm Oral Feeding Readiness Scales (11). In this study, the oromotor therapy group demonstrated better oral motor skills, transitioning to full oral feeding, and a better transition to breastfeeding after discharge (Balcı et al.).

During the transition to oral feeding in preterm infants, the mode of feeding, in addition to oromotor interventions, plays a crucial role. Breast milk is associated with the protection from necrotizing enterocolitis, retinopathy of prematurity, and bronchopulmonary dysplasia, and also leads to better neurodevelopmental outcomes (12, 13). Several studies have shown that exclusive breast milk feeding improves tolerance to enteral feeding vs. formula feeding. However in daily practice, infants who are breastfed often receive formula as part of their intake due to insufficient availability of their mother's milk or donor human milk (12, 13). In a recently published study, Kumar et al. analyzed the relationship between feeding mode and volumes with feeding intolerance in exclusively oral formula-fed or breast-fed and hybrid fed infants (formula feeds supplement in addition to breast milk) in infants born at ≥35 weeks in the first 24 h of life. Emesis, which is one of the signs of feeding intolerance, was observed more frequently in initial formula-fed infants compared to breastfed infants, that supported breast milk is the gold standard for nutrition in both preterm and term infants. The study also demonstrated that higher volumes of feeding within in the first 24 h of life, might cause feeding intolerance. Standardizing feeding volumes, accurately recording the feeding amounts during the first 24 h of life and knowing the feeding amount in this critical period could help prevent unnecessary tests and investigations related to diagnosing feeding intolerance (Kumar et al.).

Another study investigated the impact of feeding methods on growth parameters and the potential occurrence of major gastrointestinal adverse events or morbidities in infants born via cesarean section. Results were compared among infants fed partially hydrolyzed synbiotic formula, standard formula or breast milk. Growth velocities during the first month of life were similar in the breast milk and partially hydrolyzed synbiotic formula groups, while weight gain in the standard formula group was significantly lower compared to the other two groups (Sahin et al.).

Partial hydrolysates are more easily digested than intact proteins (14). Studies have shown that preterm infants fed partially hydrolyzed formulas had accelerated transit times compared to those fed standard formulas (15, 16). The inclusion of prebiotics, probiotics, or a combination of both (referred to as synbiotics) in infant formula has led to a stool microbiota profile more similar to that of infants fed breast milk (17). This approach may provide benefits for nutrient digestion and absorption, as well as to the development of the immune system. Wang et al. (18) investigated growth and gastrointestinal tolerance in infants fed a partially hydrolyzed synbiotic formula compared with an intact protein synbiotic formula. They demonstrated that a partially hydrolyzed synbiotic formula supports adequate growth, is well tolerated, and is suitable for use in healthy, term-born infants.

Despite all these feeding methods, breastfeeding within the first hours of life is crucial for providing optimal nutrition. The rate of exclusive breastfeeding of babies within first 6 months after birth is currently far below the target (19). The breast crawl is one of the method that makes breastfeeding more natural by tapping into the neonate's instinct to find milk autonomously. Breast crawl refers to the natural crawling toward the mother's breast; in this process of skin-to-skin contact between mother and infant without external interference, the neonate locates the nipple and self-attaches for the first feeding. Pang et al. investigated the long-term effects of breast crawl on breastfeeding. In this study, the overall success rate of the first feeding was 89.4% in the successful group. The initiation time, the duration of the first feeding and the lactation initiation were earlier in this group and they showed improvement in the rate of breastfeeding within the 48 h of delivery (Pang et al.). However, further studies are needed to assess the long-term effects of breast crawl on breastfeeding.

Family members' support and active participation to the feeding process are important in improving growth and health outcomes in infants. The paternal education level plays a significant role in breastfeeding initiation and appears to be a predictor of infant health outcomes (20). Studies have shown that, particularly in low- and middle-income countries, fathers' involvement in feeding young children and supporting all aspects of feeding practices can lead to improved nutritional outcomes (21, 22). Hailu et al. investigated feeding practices for sick children (infant or young child with either common childhood illnesses or other illnesses and seeking treatment) and associated factors among mothers with children under 2 years of age in southern Ethiopia. The study found that good feeding practices were linked to being an urban resident and being employed, as well as having antenatal and postnatal care visits. Moreover, paternal involvement in feeding sick children was significantly associated with better feeding practices (Hailu et al.).

This research topic highlights the prevalence of feeding difficulties in preterm infants, the importance of implementing targeted support interventions to promote breastfeeding during hospitalization and after discharge, and the significance of parent-infant interaction, all of which contribute to the regulation of feeding behaviors later in life. Early recognition of feeding difficulties in preterm infants, especially during NICU follow-up, is crucial for ensuring appropriate management. Addressing these difficulties requires a multidisciplinary approach, including NICU follow-up, education, and support.

## References

[1] Dodrill P Gosa MM. Pediatric dysphagia: physiology, assessment, and management. Ann Nutr Metab. (2015) 66:24–3. 10.1159/000381372 26226994

[2] Rodriguez Gonzalez P Perez-Cabezas V Chamorro-Moriana G Ruiz Molinero C Vazquez-Casares AM Gonzalez-Medina G. Effectiveness of oral sensory-motor stimulation in premature infants in the neonatal intensive care unit (NICU) systematic review. Children. (2021) 8:758. 10.3390/children8090758 34572190 PMC8465336

[3] Lau C. Development of infant oral feeding skills: what do we know? Am J Clin Nutr. (2016) 103:616–21. 10.3945/ajcn.115.109603 26791183 PMC4733254

[4] Merritt TA Pillers D Prows SL. Early NICU discharge of very low birth weight infants: a critical review and analysis. Semin Neonatol. (2003) 8:95–115. 10.1016/S1084-2756(02)00219-1 15001147

[5] McGrath JM Braescu AV. State of the science: feeding readiness in the preterm infant. J Perinat Neonatal Nurs. (2004) 18:353–68. 10.1097/00005237-200410000-00006 15646306

[6] Ince DA Ecevit A Acar BO Saracoglu A Kurt A Tekindal MA Noninvasive evaluation of swallowing sound is an effective way of diagnosing feeding maturation in newborn infants. Acta Paediatr. (2014) 103(8):e340–8. 10.1111/apa.12686 24814215

[7] Pineda R Prince D Reynolds J Grabill M Smith J. Preterm infant feeding performance at term equivalent age differs from that of full-term infants. J Perinatol. (2020) 40(4):646–54. 10.1038/s41372-020-0616-2 32066844 PMC7117861

[8] Neu J Zhang L. Feeding intolerance in very-low-birthweight infants: what is it and what can we do about it? Acta Paediatr Suppl. (2005) 94:93–9. 10.1111/j.1651-2227.2005.tb02162.x 16214773

[9] Cortez J Makker K Kraemer DF Neu J Sharma R Hudak ML. Maternal milk feedings reduce sepsis, necrotizing enterocolitis and improve outcomes of premature infants. J Perinatol. (2018) 38:71–4. 10.1038/jp.2017.149, 29048409

[10] Barr PA Mally PV Caprio MC. Standardized nutrition protocol for very low- birth-weight infants resulted in less use of parenteral nutrition and associated complications, better growth, and lower rates of necrotizing enterocolitis. JPEN J Parenter Enteral Nutr. (2018) 43:540–9. 10.1002/jpen.1453 30414179

[11] Aykanat Girgin B Gözen D Uslubaş R Bilgin L. The evaluation of oral feeding in preterm infants: Turkish validation of the early feeding skills assessment tool. Turk Arch Pediatr. (2021) 56(5):440–6. 10.5152/TurkArchPediatr.2021.21008 35110111 PMC8849411

[12] Boyd CA Quigley MA Brocklehurst P. Donor breast milk versus infant formula for preterm infants: systematic review and meta-analysis. Arch Dis Child Fetal Neonatal Ed. (2007) 92:F169–75. 10.1136/adc.2005.089490 16556615 PMC2675323

[13] Henderson G Anthony MY McGuire W. Formula milk versus maternal breast milk for feeding preterm or low birth weight infants. Cochrane Database Syst Rev. (2007) 17(4):CD002972. 10.1002/14651858 17943777

[14] Billeaud C Guillet J Sandler B. Gastric emptying in infants with or without gastro-oesophageal reflux according to the type of milk. Eur J Clin Nutr. (1990) 44:577–83. 2209513

[15] Vandenplas Y Ksiażyk J Luna MS Migacheva N Picaud JC Ramenghi LA Partial hydrolyzed protein as a protein source for infant feeding: do or don't? Nutrients. (2022) 21(14):1720. 10.3390/nu14091720 35565688 PMC9103110

[16] Picaud JC Rigo J Normand S Lapillonne A Reygrobellet B Claris O Nutritional efficacy of preterm formula with a partially hydrolyzed protein source: a randomized pilot study. J Pediatr Gastroenterol Nutr. (2001) 32:555–61. 10.1097/00005176-200105000-00012 11429516

[17] Salminen S Stahl B Vinderola G Szajewska H. Infant formula supplemented with biotics: current knowledge and future perspectives. Nutrients. (2020) 30(12):1952. 10.3390/nu12071952 32629970 PMC7400136

[18] Wang Y Li Z Wu JL Zhang L Liu M Tan M Botma A Liu M Mulder KA Abrahamse-Berkeveld M Cai W. A partially hydrolyzed formula with synbiotics supports adequate growth and is well tolerated in healthy, Chinese term infants: a double-blind, randomized controlled trial. Nutrition. (2021) 91–92:111472. 10.1016/j.nut.2021.111472 34626956

[19] Meek JY Noble L. Section on breastfeeding. Policy statement: breastfeeding and the use of human milk. Pediatrics. (2022) 150:e2022057988. 10.1542/peds.2022-057988 35921640

[20] Hackman NM Sznajder KK Kjerulff KH. Paternal education and its impact on breastfeeding initiation and duration: an understudied and often overlooked factor in U.S. breastfeeding practices. Breastfeed Med. (2022) 17(5):429–36. 10.1089/bfm.2021.0338 35180349 PMC9127829

[21] Kansiime N Atwine D Nuwamanya S Bagenda F. Effect of male involvement on the nutritional status of children less than 5 years: a cross sectional study in a rural southwestern district of Uganda. J Nutr Metab. (2017) 2017:3427087. 10.1155/2017/3427087 29348935 PMC5733895

[22] Flax VL Ouma EA Schreiner MA Ufitinema A Niyonzima E Colverson KE Engaging fathers to support child nutrition increases frequency of children’s animal source food consumption in Rwanda. PLoS One. (2023) 18:e0283813. 10.1371/journal.pone.0283813 37027367 PMC10081762

